# The complete mitochondrial genome of 3 species of allocreadiids (Digenea, Allocreadiidae): characterization and phylogenetic position within the order Plagiorchiida

**DOI:** 10.1017/S0031182024000064

**Published:** 2024-03

**Authors:** Brenda Solórzano-García, David I. Hernández-Mena, Anindo Choudhury, Gerardo Pérez-Ponce de León

**Affiliations:** 1 Escuela Nacional de Estudios Superiores unidad Mérida (ENES-Mérida, UNAM), Mérida, Yucatán, Mexico; 2 St. Norbert College, De Pere, WI, USA

**Keywords:** *Allocreadium*, *Creptotrematina*, DNA, mitogenome, Trematoda, *Wallinia*

## Abstract

Trematodes of the family Allocreadiidae are primarily found in the intestines of freshwater fishes around the world. The family includes 15 genera and c. 130 species. The last 2 decades have witnessed an increase in the genetic library of its species. Molecular data have been crucial for species delimitation and species description within Allocreadiidae and for understanding their evolutionary and biogeographical history and classification. Here, the mitogenomes of 3 species of allocreadiids were obtained using high throughput sequencing methods. Mitogenomes were compared with other members of the order Plagiorchiida to determine their molecular composition, gene rearrangement and phylogenetic interrelationships. The complete circular mitogenomes of *Allocreadium lobatum, Creptotrematina aguirrepequenoi* and *Wallinia mexicana* were 14 424, 13 769 and 13 924 bp long respectively, comprising 12 protein-coding genes, 22 transfer RNA genes, 2 ribosomal RNA genes and 2 non-coding regions. Gene arrangements were identical to other Xiphidiatan trematodes. Phylogenetic analyses using the mitogenomes revealed Allocreadiidae as a monophyletic group closely related to other members of the suborder Xiphidiata; *A. lobatum* was yielded as the sister taxon of *C. aguirrepequenoi* + *W. mexicana*. Our study increases the complete mitochondrial genome library of trematodes and strengthens our understanding of the phylogenetic relationships and classification of this parasite group.

## Graphical abstract

**Figure:**
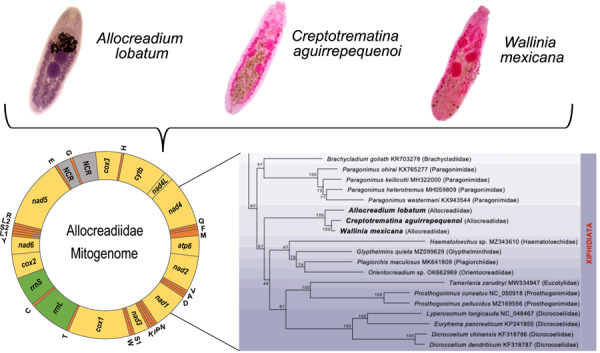

## Introduction

The family Allocreadiidae contains trematodes commonly found, as adults, in the intestine of freshwater fishes, and occasionally in snakes, salamanders and frogs (Caira and Bogéa, 2005). Adult allocreadiids typically possess an unspined tegument, a well-developed cirrus-sac, gonads in tandem and relatively large eggs. Allocreadiid metacercariae encyst in the haemocoel of aquatic arthropods; the cercariae are ophthalmoxiphidocercariae and develop in sphaeriid clams rather than in gastropods (Caira and Bogéa, 2005; Choudhury *et al*., 2007). *Allocreadium* is the type-genus and the most speciose among allocreadiids. The somewhat complex taxonomic history of the family was discussed in detail by Caira and Bogéa (2005). These authors also revised the genus composition within the family and, after discussing the doubtful validity of 13 of them, concluded at the time that Allocreadiidae comprised 14 genera. The phylogenetic position of the family within the trematode order Plagiorchiida was first tested using molecular data by Curran *et al*. (2006) and Choudhury *et al*. (2007) using 28S rDNA sequences or a combined analysis of 28S and 18S rDNA, respectively. In both analyses, allocreadiids formed a highly supported monophyletic clade with species of Callodistomidae and Gorgoderidae, which, in turn appeared as sister taxa to a clade containing representative species of Encyclometridae, Brachycoeliidae, Dicrocoelidae and Orchipedidae.

Traditional approaches based on morphology have not been useful to assess the interrelationships among members of the family, although a significant progress has been made unravelling the species delimitation, evolutionary history, classification and historical biogeography of this group of trematodes by using DNA sequences. For instance, 2 genera validated by Caira and Bogéa (2005), *Paracreptotrematina* and *Polylekithum,* were proved not to belong in Allocreadiidae (Curran *et al*., 2006, 2011; Choudhury *et al*., 2007); the genera *Margotrema* and *Wallinia* were validated as allocreadiids and not macroderoidids (Pérez-Ponce de León *et al*., 2007); furthermore, 3 of the genera considered invalid by Caira and Bogéa (2005) have been resurrected, i.e., *Acrolichanus*, *Stephanophiala* and *Megalogonia* (Atopkin *et al*., 2020; Vainutis *et al*., 2021); finally, 4 new genera have been described (*Paracreptotrema*, *Paracreptotrematoides*, *Pseudoparacreptotrema* and *Mesoamericatrema*) (Choudhury *et al*., 2006; Pérez-Ponce de León *et al*., 2016, 2020; Mendoza-Garfias *et al*., 2022). Our own account on the taxonomic composition of Allocreadiidae shows that the family currently contains 15 genera and approximately 130 species. This provides but a glimpse on the progress made on the classification scheme and species composition within the family Allocreadiidae after the revisionary work by Caira and Bogéa (2005).

The application of molecular tools has provided useful information that expands our knowledge about the species diversity of the family and their interrelationships (e.g., Faltýnková *et al*., 2020; Pérez-Ponce de León *et al*., 2020; Franceschini *et al*., 2021; Petkevičiūtė *et al*., 2023; Sokolov *et al*., 2023; Vainutis *et al*., 2023). Since the year 2000, c. 40 new species have been described following an integrative taxonomy approach, and molecular phylogenetic hypotheses have addressed aspects of the classification scheme of the group, providing more robust species delimitation criteria upon which biogeographical interpretations are made and nomenclatural changes proposed (see Atopkin *et al*., 2020; Franceschini *et al*., 2021; Vainutis *et al*., 2021). In the same period, about 30 papers generated information to enhance the genetic library of ribosomal and mitochondrial genes for allocreadiid species, particularly the 28S rDNA and the cytochrome oxidase subunit 1, *cox*1.

Complete mitochondrial genomes (mt genomes) obtained through high throughput sequencing methods have been used in the last 20 years for reconstructing the phylogenetic history of trematodes and to test the higher-level classification scheme. The use of mt genomes is based on the premise that the number of sequenced base pairs, gene order and genome arrangement may provide evidence of shared ancestry (see Littlewood *et al*., 2006), although their power to resolve deep levels of phylogenetic trees remains controversial (Pérez-Ponce de León and Hernández-Mena, 2019). Still, mt genomes have not been generated for species in the family Allocreadiidae to test their interrelationships with other trematodes. Thus, the main objective of this paper was to sequence the mt genome of 3 species of allocreadiids, namely *Allocreadium lobatum*, *Creptotrematina aguirrepequenoi* and *Wallinia mexicana*, parasites of freshwater fishes, to determine the gene content, arrangement, and composition, and to assess the phylogenetic relationships with other trematode species belonging in the order Plagiorchiida.

## Materials and methods

### Sample collection, morphological identification, and DNA extraction

Specimens of allocreadiids were sampled from the intestines of their host as follows: Specimens of *C. aguirrepequenoi*, *W. mexicana*, and *A. lobatum* were isolated from *Astyanax mexicanus* (Jalpan, Queretaro, Mexico), *Astyanax aeneus* (Matías Romero, Oaxaca, Mexico) and *Luxilus cornutus/Semotilus atromaculatus* (West Twin River, Wisconsin, USA), respectively. Some specimens were killed and relaxed with nearly boiling tap water and stored in vials with 96% ethanol for further processing (staining and mounting) and morphological identification. Trematodes were identified to species level following previous bibliographic accounts. Some other specimens were thoroughly washed in physiological saline solution and fixed in 100% ethanol for molecular analysis. To confirm the taxonomic identity, DNA was extracted from 1 specimen of each morphologically identified species and a fragment of the 28S ribosomal RNA gene was amplified as described in previous studies, using the primers 502 (5′ – CAAGTACCGTGAGGGAAAGTTGC- 3′) and 536 (5′ – CAGCTATCCTGAGGGAAAC-3′) (García-Varela and Nadler, 2005). PCR products were sequenced at the LANBIO-IBUNAM, Mexico City and a BLAST-N search (https://blast.ncbi.nlm.nih.gov/Blast.cgi) was performed.

For mt genome sequencing, genomic DNA was extracted from a pool of 5–9 individuals of each trematode species using QIAamp Fast DNA Tissue kit (Qiagen) according to the manufacturer's protocol. The dosage of the obtained DNA was determined in a microvolume spectrophotometer (NanoDrop_ND-1000).

### High throughput sequencing and assembly

Extracted genomic DNA of each trematode species was shotgun sequenced on an Illumina HiSeq 4000 with a 100x depth, and 150-bp paired-end libraries were built with Nextera adaptors at Azenta GENEWIZ (NJ, USA). Bioinformatic analysis and de-novo genome assembly of the obtained reads were also conducted by Azenta. SOAPDeNovo2 (Luo *et al*., 2012) was used on each of the samples with a minimum contig length of 1000 bp. EMBOSS tools GetOrf was then used to find the open reading frames (ORF) within the *de novo* assembled genome. The protein sequences from ORF were then annotated using Diamond BLASTp. To extract the mt genome, scaffolds were mapped onto a barcode sequence of the *cox1* gene from each allocreadiid species in this study using the software Geneious Prime v.11 with the highest sensitivity and up to 5 iterations. This initial assembly was then used to seed iterative extensions until even and thorough coverage was obtained. The final assembly was annotated with MITOS Webserver (refseq = 63, translation table = 5) (Bernt *et al*., 2013). The ORF of each protein gene was verified using Geneious, employing the invertebrate mitochondrial genetic code. Putative tRNA genes and their secondary structure were identified using ARWEN (Laslett and Canbäck, 2008) and MITOS. Circular mitochondrial genome maps were drawn using GenomeVx tool (Conant and Wolfe, 2008).

### Comparative analysis among allocreadiid mt genomes.

Contents of A + T and G + C were calculated in Geneious. AT-skew and GC-skew values were calculated using the equations AT-skew = (A- T)/(A + T) and GC-skew = (G- C)/(G + C) in each of the 12 protein-coding genes (PCGs) and the non-coding regions (NCRs). The PCG sequences were translated into their corresponding amino acid sequences using MEGA X (Kumar *et al*., 2018) and the invertebrate mitochondrial genetic code. Amino acid composition and relative synonymous codon usage (RSCU) was also estimated in MEGA X. Mutation rate (non-synonymous/synonymous, dN/dS) ratios among the 12 PCGs of the 3 newly sequenced allocreadiid mt genomes were calculated using DnaSP v.6.12 (Rozas *et al*., 2017). To estimate nucleotide diversity (π) across genes, a sliding window analysis was implemented in DnaSP using a window size of 300 bp and a step size of 30 bp, for the 12 PCGs plus the 2 rRNAs (*rrnS* and *rrnL*); values of nucleotide diversity were then plotted according to the midpoint position of each window. Nucleotide and amino acid differences were calculated using MEGA X

### Phylogenetic analyses

The newly sequenced mt genomes were used along with those of other plagiorchiid trematodes downloaded from the GenBank database to reconstruct phylogenetic trees. Analyses included species allocated in the suborders Echinostomata, Opisthorchiata and Xiphidiata. Based on previous molecular phylogenetic analyses (Pérez-Ponce de León and Hernández-Mena, 2019), *Fasciola hepatica* (AF216697) was used as an outgroup. A concatenated matrix of the 12 PCGs was built in Geneious. Previously, each gene was independently aligned in ClustalW through the GenomeNet web interface (www.genome.jp). Phylogenetic relationships were inferred using 2 datasets: the first one with the nucleotide alignment of 12 PCGs plus the 2 ribosomal genes (*rrnS* and *rrnL*), and the second with the amino acid alignment. Phylogenetic trees were constructed using the maximum likelihood (ML) method in RaxML (Stamatakis, 2014). Empirical substitution models GTR + CAT and PROTGAMMA were employed for nucleotide and amino acid data sets, respectively; bootstrap support values for nodes were estimated with 1000 replicates.

## Results and discussion

### General features of allocreadiid mitogenomes

**Figure 1.**
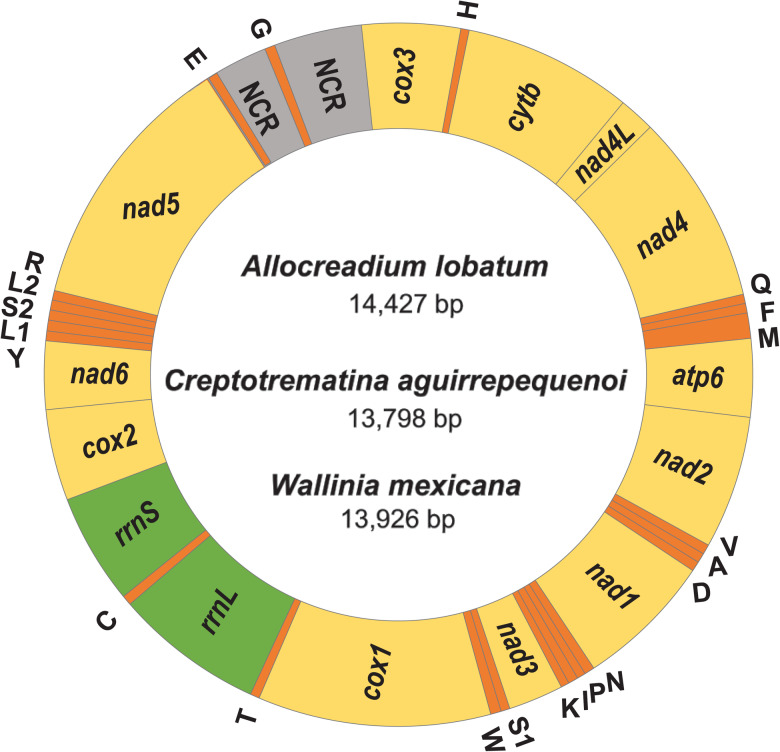
Gen map of the 3 allocreadiid mitochondrial genomes, *Allocreadium lobatum, Creptotrematina aguirrepequenoi, Wallinia mexicana.* NCR, non-coding regions.

The complete circular mt genomes of *A. lobatum*, *C. aguirrepequenoi* and *W. mexicana* were 14,427, 13,798 and 13 926 pb long, respectively (GenBank accession numbers: OR987847, OR987848, OR987849, respectively). These mt genomes were composed of 12 PCGs (*cox1 – 3*, *nad 1–6, cytb and atp6*; *atp8* gene is lacking), 2 rRNA genes (*rrnS* and *rrnL*), 22tRNAs (1 for each amino acid and 2 each for leucine and serine), and 2 non-coding regions (NCR) (Fig. 1 and Table 1). All genes were transcribed in the same direction. Total length and gene order of all 3 newly sequenced allocreadiid mt genomes corresponds to the typical arrangement observed in other species allocated in the order Plagiorchiida (Liu *et al*., 2014; Briscoe *et al*., 2016; Chang *et al*., 2016; Qian *et al*., 2018; Le *et al*., 2019; Suleman *et al*., 2019, 2020, 2021; Guo *et al*., 2022; Gao *et al*., 2023). The order of the 12PCGs seems to be highly conserved among trematodes, with the location of tRNAs as variable among species (Suleman *et al*., 2019; Gao *et al*., 2023).

**Table 1.** Comparison of the mitochondrial genome arrangement of *Allocreadium lobatum* (Al), *Creptotrematina aguirrepequenoi* (Cp.) and *Wallinia mexicana* (Wa)

Genes	Star position	End position	Length	Initiation codon	Stop codon	Anticodon	NN similarity	AA similarity
	Al/Cp/Wa	Al/Cp/Wa	Al/Cp/Wa	Al/Cp/Wa	Al/Cp/Wa			
*cox3*	1/1/1	645/645/645	645/645/645	ATG/ATG/ATG	TAA/TAG/TAA		82.4	81.2
*trnH*	661/663/663	717/716/716	57/54/54			GTG		
*cytb*	724/725/725	1833/1834/1834	1110/1110/1110	ATG/ATG/ATG	TAG/TAG/TAG		90.1	92.8
*nad4L*	1867/1868/1868	2103/2104/2104	237/237/237	ATG/ATG/GTG	TAG/TAG/TAG		82.1	79.7
*nad4*	2064/2065/2065	3320/3321/3345	1257/1257/1281	ATG/ATG/GTG	TAA/TAA/TAA		84.2	80.9
*trnQ*	3321/3323/3346	3381/3383/3406	61/61/61			TTG		
*trnF*	3383/3385/3408	3445/3447/3470	63/63/63			GAA		
*trnM*	3346/3448/3471	3512/3512/3535	67/72/65			CAT		
*atp6*	3513/3513/3536	4028/4028/4051	516/516/516	ATG/ATG/ATG	TAG/TAA/TAG		75.6	80.8
*nad2*	4035/4035/4052	4913/4901/4903	879/867/852	ATG/ATG/ATG	TAG/TAG/TAA		79.2	75.3
*trnV*	4914/4914/4918	4978/4979/4980	65/66/63			TAC		
*trnA*	4988/4982/4983	5051/5055/5044	64/63/62			TGC		
*trnD*	5056/5052/5053	5120/5116/5117	65/65/65			GTC		
*nad1*	5121/5117/5118	6026/6022/6023	906/906/906	ATG/ATG/ATG	TAG/TAG/TAG		91.5	90.1
*trnN*	6036/6032/6033	6102/6098/6099	67/67/67			GTT		
*trnP*	6103/6099/6100	6157/6164/6166	56/66/66			TGG		
*trnI*	6166/6165/6167	6229/6228/6230	64/64/64			GAT		
*trnK*	6237/6232/6234	6300/6299/6299	64/68/66			CTT		
*nad3*	6304/6301/6303	6663/6660/6662	360/360/360	ATG/ATG/ATG	TAG/TAA/TAG		75.8	78.5
*trnS1*	6669/6666/6666	6729/6724/6724	61/59/59			GCT		
*trnW*	6731/6726/6726	6791/6791/6791	61/66/66			TCA		
*cox1*	6810/6798/6798	8336/8336/8336	1527/1539/1539	TGA/ATG/ATG	TAG/TAG/TAG		79.7	85.1
*trnT*	8337/8337/8337	8402/8402/8402	66/66/66			TGT		
*rrnL*	8404/8403/8403	9377/9369/9383	974/966/980					
*trnC*	9381/9386/9387	9446/9443/9444	66/58/57			GCA		
*rrnS*	9450/9448/9448	10178/10174/10180	729/727/755					
*cox2*	10202/10197/10203	10801/10796/10802	600/600/600	ATG/ATG/ATG	TAA/TAA/TAG		81.8	80.7
*nad6*	10803/10798/10804	11252/11247/11253	450/450/453	ATG/GTG/GTG	TAA/TAG/TAA		83.0	85.0
*trnY*	1125611253/11257	11319/11315/11320	65/63/64			GTA		
*trnL1*	11325/11321/11342	11388/11383/11412	64/63/71			TAG		
*trnS2*	11396/11381/11413	11453/11445/11478	58/65/66			TGA		
*trnL2*	11463/11450/11480	11528/11515/11547	66/64/68			TAA		
*trnR*	11529/11519/11549	11592/11577/11614	64/59/66			TCG		
*nad5*	11625/11586/11677	13199/13160/13251	1596/1575/1599	ATT/ATT/ATT	TAA/TAA/TAA		84.2	87.1
*trnE*	13225/13187/13276	13294/13250/13347	70/64/72			TTC		
NCR	13295/13251/13348	13783/13427/13514	489/177/167					
*trnG*	13784/13429/13515	13852/13497/13583	69/70/69			TCC		
NCR	13853/13498/13584	14427/13798/13926	572/301/397					

Similarity values are expressed in percentage. NN, nucleotides; AA, amino acids.

Intergenic spacers ranged from 1–34 bp in *A. lobatum* and *C. aguirrepequenoi*, and from 1–63 bp in *W. mexicana*. A 40 bp overlap between *nad4L* and *nad4* was present in the 3 mt genomes. This is consistent with some species of xiphidiatan trematodes such as *Paragonimus heterotremus*, *P. ohirai*, *Prosthogonimus* spp., *Tamerlania zarudnyi* and *Plagiorchis maculosus* (Qian *et al*., 2018; Le *et al*., 2019; Suleman *et al*., 2019, 2021; Guo *et al*., 2022). However, other xiphidiatans (e.g., *Dicrocoelium dendriticum*, *D. chinensis* and *Paragonimus westermani*) show an overlap of just 1– 14 bp (Biswal *et al*., 2014; Liu *et al*., 2014).

The *rrnL* gene was located between *trnT* and *trnC*, while the *rrnS* gene was located between *trnC* and *cox2* in the 3 allocreadiid species. The length of the 22 tRNA genes was 1403 bp in *A. lobatum*, 1404 bp in *C. aguirrepequenoi*, and 1,427 in *W. mexicana*, whereas individual tRNA gene lengths ranged from 54 to 72 bp (Table 1). The secondary structures predicted for the tRNA genes were similar among the 3 allocreadiids. Twenty of the tRNAs in *C. aguirrepequenoi* and *W. mexicana,* along with 18 tRNAs in *A. lobatum* can be folded into the conventional cloverleaf secondary structure. The D-arm is absent in the tRNA for Serine 1 (*trnS1*) in the 3 species, in the *trnS2* only in *A. lobatum,* and in the tRNA for Cysteine in *C. aguirrepequenoi* and *W. mexicana* (Fig S1). The tRNAs for Proline (*trnP*) and Lysine (*trnK*) are lacking the T-arm in *A. lobatum* (Fig S1). The lack of D-arm in the Serine 1 tRNAs is consistent with previous reports on mt genomes of other trematodes (Chang *et al*., 2016; Le *et al*., 2019; Suleman *et al*., 2021; Guo *et al*., 2022). Likewise, tRNAs with non-canonical structures have been observed in different organisms including mammals, nematodes and arthropods, where either T-arm, D-arm or both could be missing (Krahn *et al*., 2020).

The 3 species contain 2 NCRs between *trnE* and *cox3*, separated by *trnG* (Fig. 1). The shorter NCR was found between *trnE* and *trnG* and varies in size from almost 500 bp in *A. lobatum* to 167 bp in *W. mexicana*; the longer NCR was located between *trnG* and *cox3* and ranged from 300 bp in *C. aguirrepequenoi* to 580 bp in *A. lobatum*. The pattern of 2 NCRs, one shorter than the other, has also been observed in other plagiorchiida including *Lyperosomum longicauda, T. zarudnyi*, *Paragonimus ohirai*; however, these are located between *trnG* and *cox3*, separated by *trnE* (Le *et al*., 2019; Suleman *et al*., 2020, 2021). This arrangement differs in *Prosthogonimus* spp. and *Plagiorchis maculosus* since both species present only 1 long NCR (Suleman *et al*., 2019; Guo *et al*., 2022), indicating that the length, arrangement and composition of the NCRs are not ubiquitous traits in Plagiorchiida mt genomes.

### Comparative analysis of allocreadiid mitogenomes

**Figure 2.**
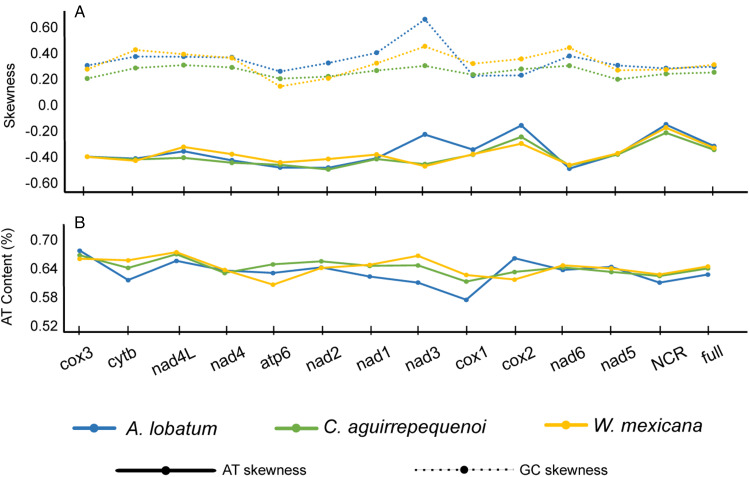
The nucleotide skewness (A) and AT content (B) of 3 species of Allocreadiids mitochondrial genomes (*Allocreadium lobatum, Creptotrematina aguirrepequenoi Wallinia mexicana).*

Gene order of the mt genome was the same among the 3 species of allocreadiids (Fig. 1); likewise, the size of each gene was similar. The rank order of the 12 PCGs by length was: *nad5 > cox1> nad4 > cytb > nad1 > nad2 > cox3 > cox2 > atp6 > nad6 > nad3 > nad4L* (Table 1). Nucleotide composition of the 3 mt genomes was biased towards A and T, with and overall AT content of 63.5% in *A. lobatum*, 64.7% in *C. aguirrepequenoi,* and 65.1% in *W. mexicana* (Fig. 2), as observed in most trematodes (Gao *et al*., 2023). A + T contents *of cytb, nad4L, nad1, nad3*, *nad6* and *cox1* were the lowest in *A. lobatum*, but the highest in *W. mexicana*; the opposite was true for *cox3* and *cox2*, with the highest A + T content in *A. lobatum.* The genes *atp6* and *nad2* showed highest A + T content in *C. aguirrepequenoi* and the lowest values in *W. mexicana* (Fig. 2). The AT-skew values in the *A. lobatum* mitogenome ranged from −0.16 (*cox2*) to −0.50 (*nad6*), the GC-skew values ranged from 0.23 (*cox1* and *cox2*) to 0.67 (*nad3*). In *C. aguirrepequenoi* the AT-skew values ranged from −0.25 (*cox2*) to −0.50 (*nad2*), the GC-skew values ranged from 0.20 (*nad5*) to 0.31 (*nad3, nad4L, nad6*). Finally, for *W. mexicana* AT-skew values ranged from −0.30 (*cox2*) to −0.48 (*nad3*), and GC-skew values ranged from 0.15 (*atp6*) to 0.46 (*nad3*) (Fig. 2).

**Figure 3.**
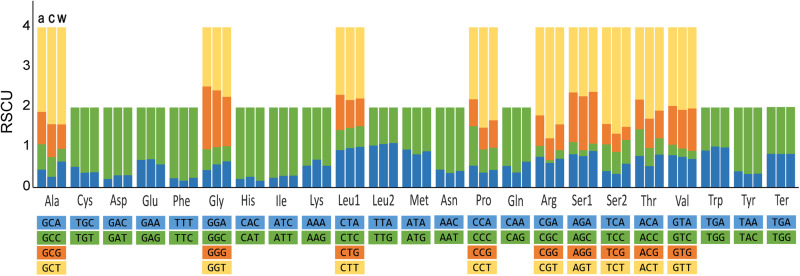
Relative synonymous codon usage (RSCU) of 12 protein coding genes of the 3 allocreadiid mt genomes: a) *Allocreadium lobatum,* c) *Creptotrematina aguirrepequenoi;* w) *Wallinia mexicana.*

The most common start codon among the 12 PCGs of the 3 allocreadiid mt genomes was ATG, with a frequency of 10/12 in *A. lobatum* and *C. aguirrepequenoi,* and 8/12 in *W. mexicana*. The TAG was the predominant stop codon in *A. lobatum* (8/12), as well as in *C. aguirrepequenoi* and *W. mexicana* (7/12) (Table 1). There were 3341, 3342 and 3,328 amino acids in 12 PCGs of *A. lobatum*, *C. aguirrepequenoi* and *W. mexicana,* respectively; the RSCU is shown in Fig. 3. In the 3 mt genomes, the most frequent amino acid was Leu (16.2–16.8%), followed by Phe (11.5–11.6%), Ser (11.1–11.4) and Val (9.2–9.6%). The least frequent amino acid was Gln (0.8–0.9%).

**Figure 4.**
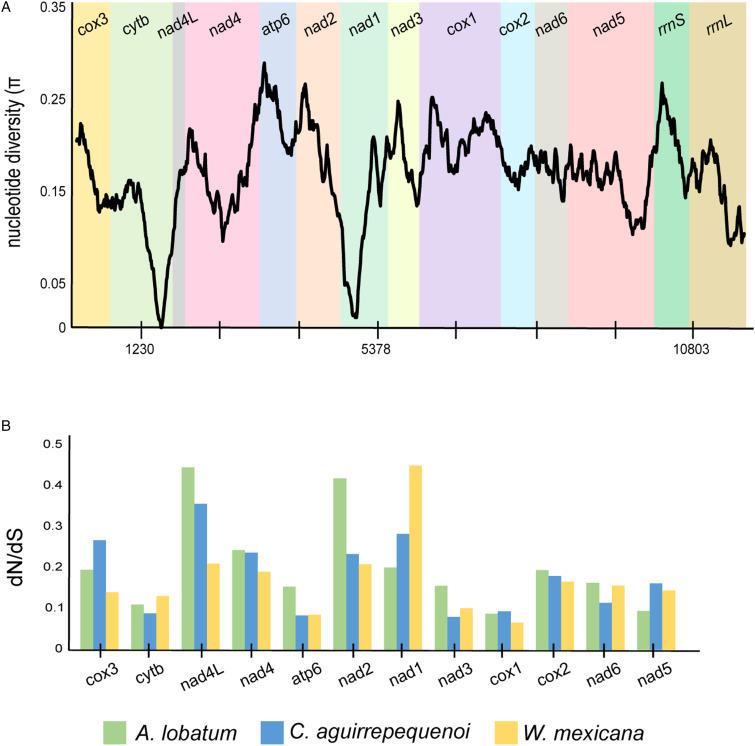
Nucleotide variation across *Alocreadium lobatum*, *Creptotrematina aguirrepequenoi* and *Wallinia mexicana* mt genomes. (A) Sliding window analysis of the 12 PCGs plus *rrnS* and *rrnL*. The black line represents nucleotide variation in a window of 300 bp (step size = 10 bp, with the value inserted at its mid-point). (B) Ratios of non-synonymous to synonymous (dN/dS) substitution rates calculated from individual protein-coding genes of 3 newly allocreadiid mt genomes.

Overall nucleotide similarity for the 12 PCGs among the 3 allocreadiid mt genomes was 75.5%, whereas amino acids showed 61.6% overall similarity. Genes with high nucleotide diversity among allocreadiids were *atp6 > nad3 > nad2> rrnS > cox1*, which is consistent with previous studies using sliding window analysis in members of Plagiorchiida (Table 1). To determine the highly conserved and variable mitochondrial genes among the 3 allocreadiid trematodes, a sliding window analysis was conducted by the concatenated nucleotide sequence of 12 PCGs plus the 2 *rrn* genes. Nucleotide diversity (π) among the 3 mt genomes ranged from 0.004 to 0.29; *atp6* (π = 0.241) was the most variable gene, while *cytb* (π = 0.10) and *nad1* (π = 0.09), showed low sequence variation (Fig. 4). *Cox 1, rrnS* and *rrnL* show intermediate molecular variation (π = 0.19, 0.21 and 0.15, respectively).

Analysis of mutation rates among the 12 PCGs of the 3 allocreadiid mt genomes showed that all PCGs were under purifying selection (dN/dS < 1) (Fig. 4); this coincides with previous estimations for other trematode species (Suleman *et al*., 2020; An *et al*., 2022). The highest non-synonymous substitution rate (dN) was observed in *nad4L* and *nad1,* while *cox1* gene showed the lowest dN/dS value, followed by *atp6*, indicating a higher synonymous variation in these genes among allocreadiids.

These results plus the distribution of nucleotide diversity among genes indicate *cox1* and *atp6* as useful genetic markers for genetic lineage determination and species identification studies in the family Allocreadiidae. It has been observed in other trematode mt genomes that *atp6* and *nad2* genes evolve faster than *cox1*, suggesting that these genes may also serve as genetic markers to capture genetic variation among closely related trematode species or even populations (Suleman *et al*., 2020).

### Phylogenetic relationships of Allocreadiidae

**Figure 5.**
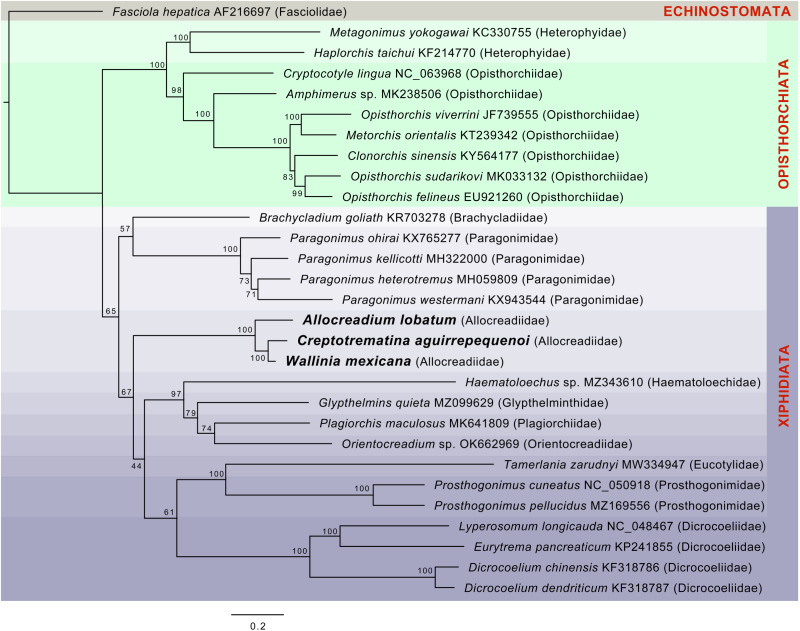
Maximum likelihood phylogenetic tree obtained with the nucleotide dataset of 12 protein-coding genes and 2 regions of the ribosomal RNA. Species in the suborder Xiphidiata are framed in purple shade. Species in Opisthorchiata are framed in green shade. The name of the species is followed by the mt genome accession number in the Genbank dataset, and in parentheses the family to which it belongs. The species sequenced in this study are highlighted in bold. Numbers near the nodes are the Bootstrap support values.

**Figure 6.**
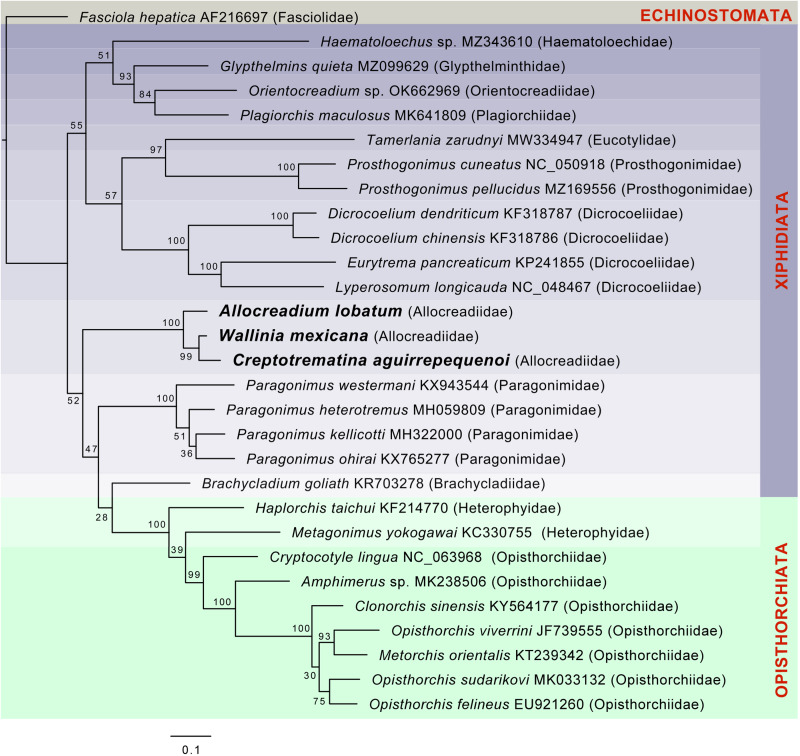
Maximum likelihood phylogenetic tree obtained the amino acids translated of 12 PCG's. Species in the suborder Xiphidiata are framed in purple shade. Species in Opisthorchiata are framed in green shade. The name of the species is followed by the mt genome accession number in the Genbank dataset, and in parentheses the family to which it belongs. The species sequenced in this study are highlighted in bold. Numbers near the nodes are the Bootstrap support values.

The ML phylogenetic trees using either nucleotides or amino acids recovered Allocreadiidae as a highly supported monophyletic group (Figs 5, 6), with *C. aguirrepequenoi* clustered together with *W. mexicana*, and these 2 with *A. lobatum*. In both analyses, allocreadiids appeared as sister taxa of other species included in the suborder Xiphidiata; this is consistent with results obtained through phylogenetic analyses based on 18S and 28S rRNA genes (Pérez-Ponce de León and Hernández-Mena, 2019), in which Allocreadiidae was recovered as a monophyletic group within the suborder Xiphidiata. Figure 5 depicts the phylogenetic tree inferred from the concatenated nucleotide sequences of 12 PCGs and 2 *rrn* for the same dataset; here the family Allocreadiidae was recovered as the sister group of a clade containing Haematoloechidae, Glypthelminthidae, Plagiorchiidae and Orientocreadiidae, plus a clade comprising Eucotylidae, Prosthogonimidae and Dicrocoeliidae. In the phylogenetic tree inferred with amino acids (Fig. 6), Allocreadiidae appeared as an early divergent clade with respect to a clade containing paragonimids plus brachycladiids (both members of Xiphidiata in the current classification, see Pérez-Ponce de León and Hernández-Mena, 2019), and all of them as sister taxa of 2 families of Opisthorchiata (Fig. 6), although these relationships are poorly supported by bootstrap values.

In the current study, phylogenetic analyses of mt genomes of 28 species of trematodes allocated in 12 families using either nucleotides or amino acids, yielded contrasting results about the monophyly of Xiphidiata, since the nucleotide tree yielded the suborder as monophyletic, whereas the amino acids tree yielded Xiphidiata paraphyletic. Our results are in contrast with Suleman *et al*. (2021) who found consistent results of the paraphyly of Xiphidiata using either nucleotide or amino acid sequences; Suleman *et al*. (2021) used the mt genomes of 30 species of trematodes allocated in 14 families. Likewise, Pérez-Ponce de León and Hernández-Mena (2019) also found Xiphidiata as paraphyletic, and the suborder Haploporata was proposed for keeping the former as monophyletic. Other mt genome phylogenetic analyses using amino acids for building the trees have consistently shown the paraphyly of Xiphidiata (Guo *et al*., 2022; Gao *et al*., 2023). In the most recently published study, Atopkin *et al*. (2023) used a comprehensive dataset of 61 mitogenomes of trematodes and corroborated Xiphidiata as paraphyletic, although their dataset was not analysed using nucleotides. Apparently, interrelationships between paragonimids and dicrocoeliids as members of the superfamily Gorgoderoidea is problematic even though both have been considered as members of Xiphidiata. Interestingly, Suleman *et al*. (2021) and Atopkin *et al*. (2023) found that Paragonimidae plus Brachycladiidae are nested with Opisthorchiidae and Heterophyidae. The amino acid phylogenetic tree in our study uncovered the same relationships, although Allocreadiidae appeared as the sister clade to these 4 families. The number and selection of taxa representative of different families, and the molecular data employed (nucleotides or amino acids), seem to exert an effect when assessing interrelationships of trematodes using mt genomes. As shown in our study, a shorter number of terminals, and a different selection of taxa may yield contrasting results.

There is no doubt that the discordant phylogenies of mt genomes obtained when using nucleotide or amino acid data, or through different inference methods, and the constant changes in the interrelationships of higher taxa when adding newly sequenced mt genomes is posing new challenges to trematode classification. In our opinion, 3 aspects must be considered to fully assess the value of mt genomes in reconstructing the phylogenetic relationships, and in elucidating higher-level interrelationships of this group of parasitic platyhelminthes. These aspects include the number of taxa sampled, outgroup selection, and use of nucleotide or amino acids. These remain key elements that need to be assessed to produce the most natural classification scheme of digenean trematodes using modern high throughput sequencing technologies.

## Supporting information

Solórzano-García et al. supplementary material Solórzano-García et al. supplementary material

## Data Availability

Mitogenome sequence data is available on the NCBI GenBank database.

## Supplementary material

The supplementary material for this article can be found at https://doi.org/10.1017/S0031182024000064.
