# Supplementary material for: The complete mitochondrial genome of 3 species of allocreadiids (Digenea, Allocreadiidae): characterization and phylogenetic position within the order Plagiorchiida
Source: Parasitology. 2024 Jan 15;151(3):309–18. doi: 10.1017/S0031182024000064 (PMC11007277; doi:10.1017/S0031182024000064)
Supplement: Solórzano-García et al. supplementary material [file S0031182024000064sup001.pdf]

Fig S1. Secondary structure of tRNAs from the three allocreadiid species, listed in order of appearance within the mt-genome.

● Non-identical in the three allocreadiid species

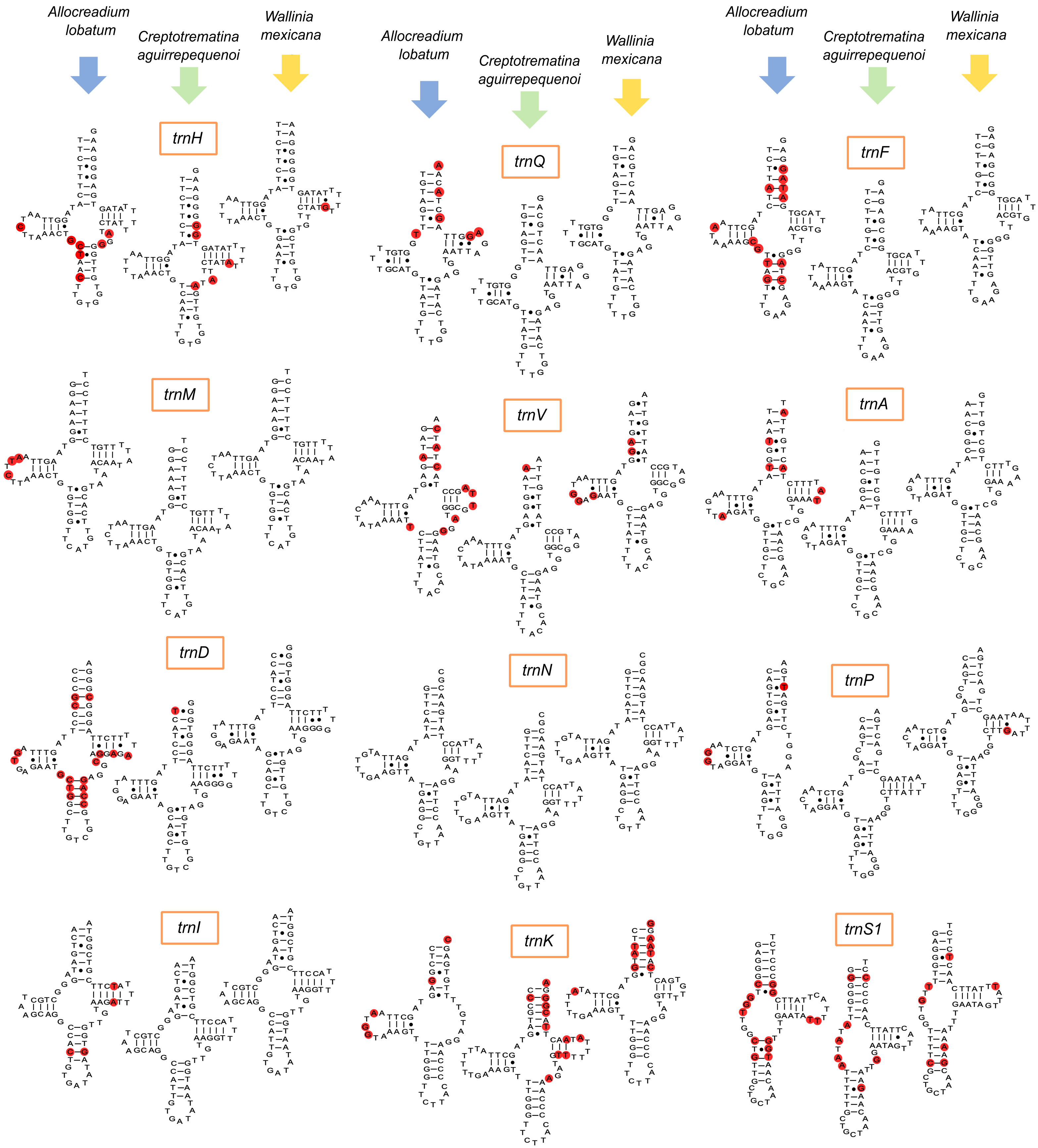

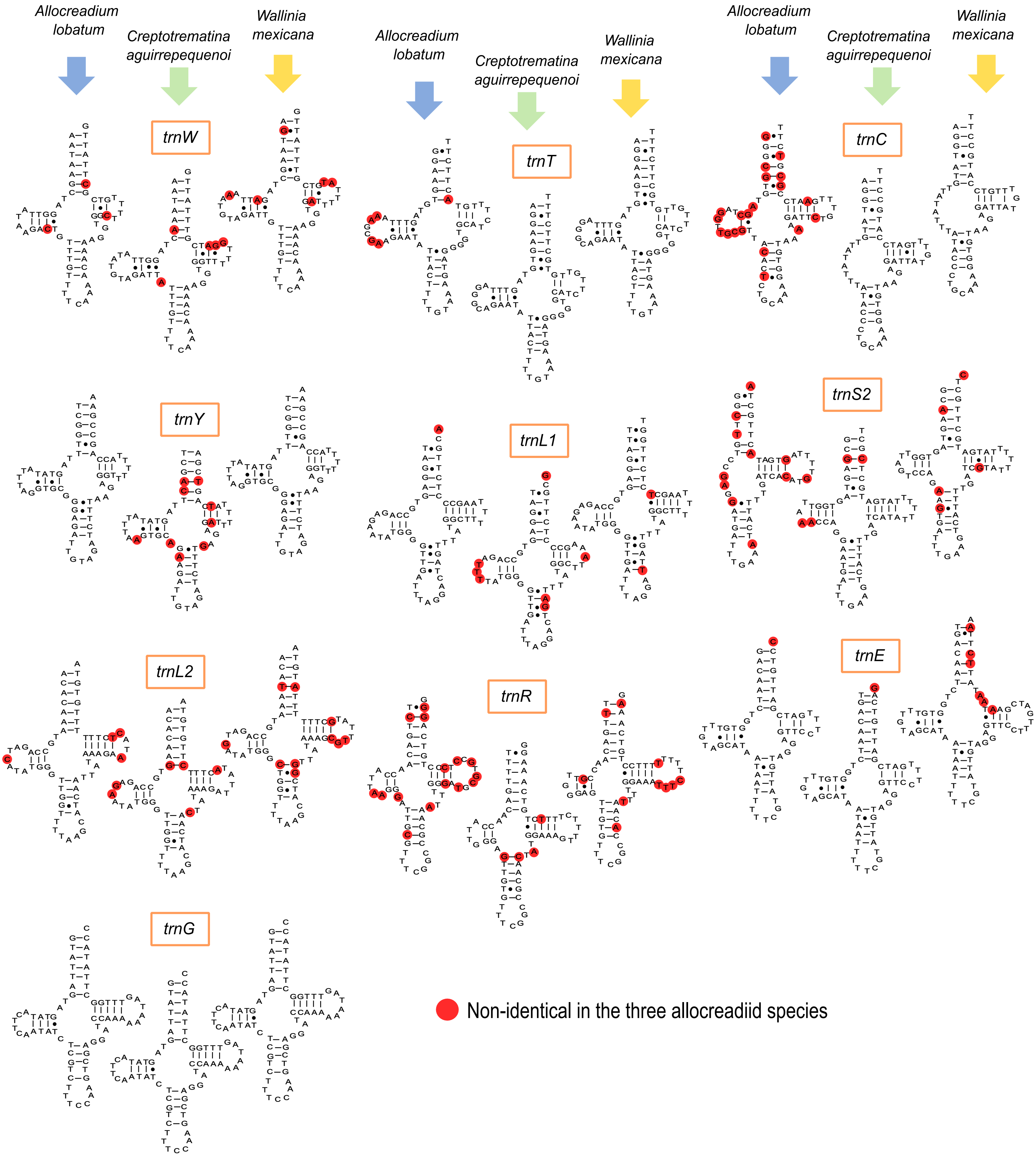

Allocreadium lobatum
Creptotrematina aguirrepequenoi
Wallinia mexicana
trnW
trnT
trnC
trnY
trnL1
trnS2
trnL2
trnR
trnE
trnG
Non-identical in the three allocreadiid species
